# Pharmacokinetic and pharmacodynamic evaluation of various vasopressin doses and routes of administration in a neonatal piglet model

**DOI:** 10.1038/s41598-024-74188-9

**Published:** 2024-10-04

**Authors:** Marwa Ramsie, Po-Yin Cheung, Megan O’Reilly, Tze-Fun Lee, Georg M. Schmölzer

**Affiliations:** 1 Centre for the Studies of Asphyxia and Resuscitation, Neonatal Research Unit, Royal Alexandra Hospital, 10240 Kingsway Avenue NW, Edmonton, AB T5H 3V9 Canada; 2 Department of Pediatrics, University of Alberta, Edmonton, AB Canada

**Keywords:** Infants, Newborn, Neonatal resuscitation, Vasopressin, Pharmacokinetics, Pharmacodynamics, Paediatric research, Preclinical research

## Abstract

Epinephrine is the only recommended vasopressor during neonatal cardiopulmonary resuscitation. However, there are concerns about the potential adverse effects of epinephrine, which might hamper efficacy during cardiopulmonary resuscitation. An alternative might be vasopressin, which has a preferable adverse effect profile, however, its optimal dose and route of administration is unknown. We aimed to compare the pharmacodynamics and pharmacokinetics of various vasopressin doses administered via intravenous (IV), intraosseous (IO), endotracheal (ETT), and intranasal (IN) routes in healthy neonatal piglets. Forty-four post-transitional piglets (1–3 days of age) were anesthetized, intubated via a tracheostomy, and randomized to receive vasopressin via intravenous (control), IO, ETT, or IN route. Heart rate (HR), arterial blood pressure, carotid blood flow, and cardiac function (e.g., stroke volume, ejection fraction) were continuously recorded throughout the experiment. Blood was collected prior to drug administration and throughout the observation period for pharmacodynamics and pharmacokinetic analysis. Significant changes in hemodynamic parameters were observed following IO administration of vasopressin while pharmacokinetic parameters were not different between IV and IO vasopressin. Administration of vasopressin via ETT or IN did not change hemodynamic parameters and had significantly lower maximum plasma concentrations and systemic absorption compared to piglets administered IV vasopressin (*p* < 0.05). The IV and IO routes appear the most effective for vasopressin administration in neonatal piglets, while the ETT and IN routes appear unsuitable for vasopressin administration.

**Supplementary Information:**

The online version contains supplementary material available at 10.1038/s41598-024-74188-9.

## Introduction

Neonatal cardiac arrest is primarily caused by perinatal asphyxia, and ~ 0.1% of term and up to 15% of preterm infants at birth will require resuscitation with high-quality chest compressions and a vasopressor (i.e. epinephrine) for survival^1,2^. The current Neonatal Consensus of Science and Treatment Recommendation (CoSTR) recommends epinephrine at a dose of 0.01–0.03 mg/kg, preferably given intravenous (IV) or intraosseous (IO) every 3–5 min during cardiopulmonary resuscitation (CPR)^3^. Epinephrine may also be given via an endotracheal tube (ETT) at a dose of 0.05–0.1 mg/kg^3^. Epinephrine is currently the only recommended vasopressor during CPR, however, epinephrine increases myocardial oxygen demand, inhibits hemodynamic responses (e.g., aggravated arterial hypertension following return of spontaneous circulation (ROSC), reduced efficacy during respiratory and metabolic acidosis, and microcirculation impairment)^4–8^.

Vasopressin, an antidiuretic hormone, might be an alternative to epinephrine, as its efficacy is not impaired during metabolic or respiratory acidosis^3,4,9^. Subgroup analysis of randomized trials of adults with out-of-hospital cardiac arrest reported that adults with asystolic arrest and treated with vasopressin had significantly higher rates of survival to hospital admission (29% vs. 20%, *p* = 0.02) and discharge (5% vs. 2%, *p* = 0.04) compared to receiving epinephrine^10^. Similarly, in a case series of four pediatric patients’ vasopressin at 0.4IU/kg/dose IV administered as rescue therapy, achieved ROSC in 3/4 children, 2/4 survived > 24 h, and 1/4 survived to hospital discharge^11^. Furthermore Duncan et al. reported that 5% of 1,293 pediatric patients received vasopressin during in-hospital cardiac arrest^12^, and although they had significantly longer duration of cardiac arrest (median 37 vs. 24 min, *p* = 0.004) and longer time to ROSC, their survival at 24 h or at discharge was not different to patients receiving epinephrine^12^. A recent feasibility study compared vasopressin (0.8 IU/kg) after an initial epinephrine dose in patients < 18 years of age (*n* = 10) to ≥ 2 doses of epinephrine^13^. Patients who received vasopressin had increased 24 h survival (80% vs. 30%, odds ratio (OR) (95%CI) 9.3 (1.5–57.7)), with no difference in time to ROSC, survival to hospital discharge, and neurologic status at discharge^13^. Therefore vasopressin may be beneficial during neonatal CPR because in newborn infants (1) asphyxia results primarily in non-shockable rhythm, rather than ventricular fibrillation and (2) pulmonary vascular resistance is high at birth in newborns. There are no pharmacodynamics and pharmacokinetic data of vasopressin available for newborn infants. Therefore, we aimed to determine pharmacodynamics and pharmacokinetics of vasopressin administered either via IV, IO, ETT, or intranasal (IN) routes to assess the optimal dose for each route in a post-transitional piglet model.

## Methods

**Fig. 1:**
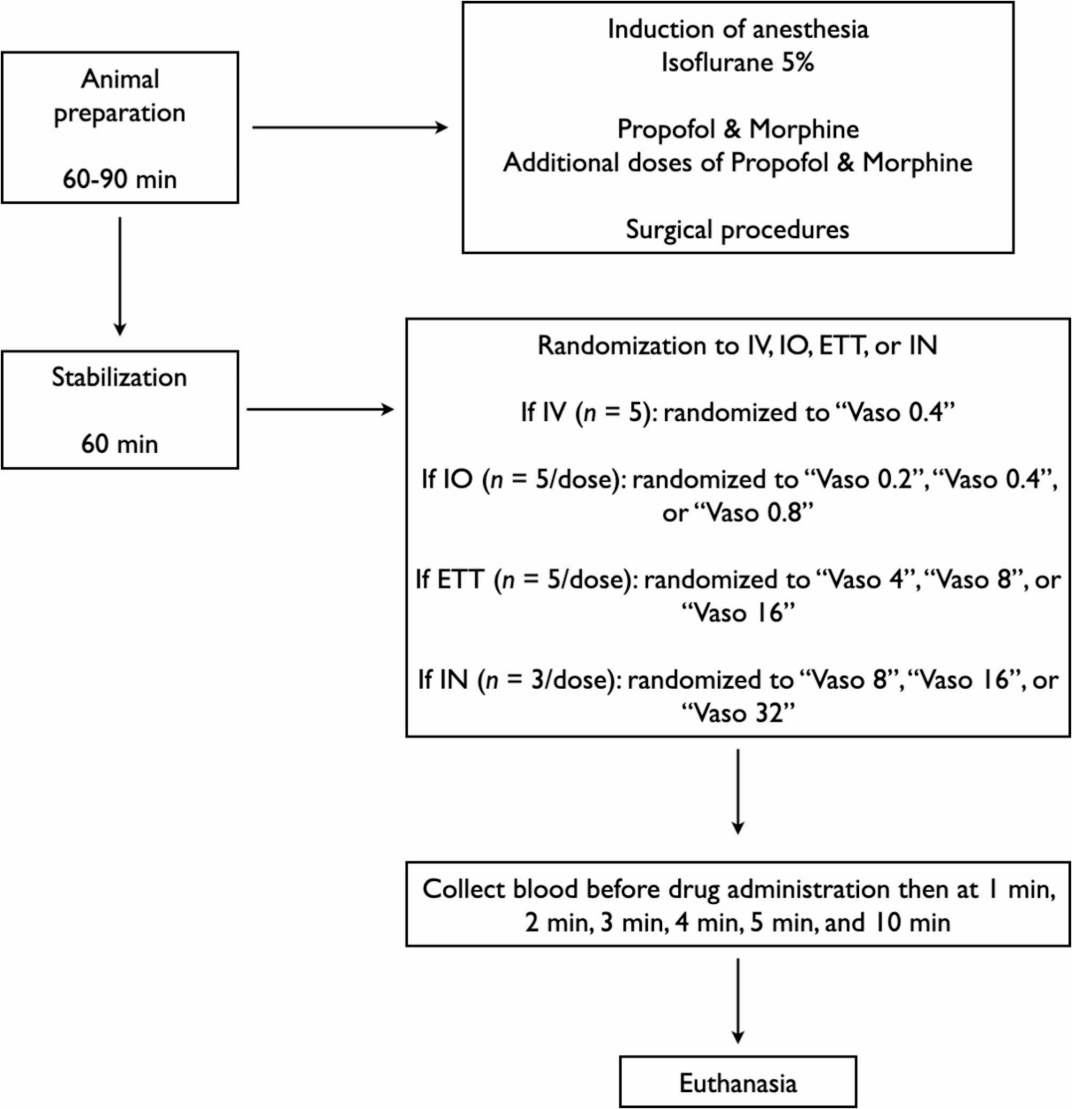
Study flow chart.

44 newborn mixed breed piglets were obtained on the day of experimentation from the University Swine Research Technology Centre located in Edmonton, Alberta, Canada. All experiments were conducted in accordance with the guidelines and approval of the Animal Care and Use Committee (Health Sciences), University of Alberta [AUP00004212], conducted and presented according to the ARRIVE guidelines^14^, conducted according to the Canadian Council of Animal Care guidelines, and registered at preclinicaltrials.eu (PCTE0000489). A graphical display of the study protocol is presented in Fig. 1.

### Inclusion and exclusion criteria

Mixed breed neonatal piglets with a current age of 1–3 days of age, weighing 2.0 kg (± 0.23 kg) were included. There was no exclusion criterion.

## Randomization

Piglets were randomly allocated to varying doses of vasopressin administered via IV, IO, ET, or IN. Allocation was block randomized with variable sized blocks using a computer-generated randomization program (http://www.randomizer.org). Sequentially numbered, sealed, brown envelopes containing the allocation were opened during the experiment (Fig. 1).

## Blinding

One investigator (TFL) opened the randomization envelope and was solely responsible for drug preparation. The content of the drug syringe was only known to TFL to conceal group allocation. All remaining team members were blinded to group allocation. The statistical analysis was blinded to group allocation and only unblinded after the statistical analysis was completed.

## Animal preparation

Piglets were instrumented as previously described with modifications^15–17^. Following the induction of anaesthesia using isoflurane, piglets were intubated via a tracheostomy, and pressure-controlled ventilation (Sechrist Infant Ventilator Model IV-100; Sechrist Industries, Anaheim, California) was commenced at a respiratory rate of 16–20 breaths/min and pressure of 20/5 cmH_2_O. Oxygen saturation was kept within 90–100%, glucose level and hydration was maintained with an intravenous infusion of 5% dextrose at 10 mL/kg/hr. During the experiment anaesthesia was maintained with intravenous propofol 5–10 mg/kg/hr and morphine 0.1 mg/kg/hr. Additional doses of propofol (1–2 mg/kg) and morphine (0.05–0.1 mg/kg) were also given as needed. The piglet’s body temperature was maintained within a normal porcine temperature range of 38.5–39.5 °C using an overhead warmer and a heating pad.

## Hemodynamic parameters

A 5-French Argyle^®^ (Klein-Baker Medical Inc. San Antonio, TX) double-lumen catheter was inserted via the right femoral vein for administration of fluids and medications. A 5-French Argyle^®^ single-lumen catheter was inserted above the right renal artery via the femoral artery for continuous arterial blood pressure monitoring in addition to arterial blood gas measurements. The right common carotid artery was also exposed and encircled with a real-time ultrasonic flow probe (2 mm; Transonic Systems Inc., Ithica, NY) to measure cerebral blood flow^15–17^. A Millar^®^ catheter (MPVS Ultra1, ADInstruments, Houston, TX) was inserted into the left ventricle via the left common carotid artery for continuous measurement of stroke volume, ejection fraction, end-diastolic and -systolic volumes, left ventricular pressure, and left ventricular contractile function (dp/dt_max_, dp/dt_min_)^19,20^.

Piglets were placed in supine position and allowed to recover from surgical instrumentation until baseline hemodynamic measures were stable (minimum of one hour). Ventilator rate was adjusted to keep the partial arterial CO_2_ between 35 and 45 mmHg as determined by periodic arterial blood gas analysis. Mean systemic arterial pressure (MAP), systemic systolic arterial pressure, heart rate, and percutaneous oxygen saturation were continuously measured and recorded throughout the experiment with a Hewlett Packard 78833B monitor (Hewlett Packard Co., Palo Alto, CA)^15–17^.

### Experimental protocol

Piglets were randomized into four routes and various vasopressin doses: (1) IV vasopressin (0.4IU/kg; *n* = 5, control), (2) IO vasopressin (*n* = 5/dose; 0.2IU/kg, 0.4IU/kg, or 0.8IU/kg), (3) ETT vasopressin (*n* = 5/dose; 4IU/kg, 8IU/kg, or 16IU/kg), (4) IN vasopressin (*n* = 3/dose; 8IU/kg, 16IU/kg, or 32IU/kg). In all groups, a 3mL saline bolus was administered immediately after the vasopressin dose^20,21^.

IV vasopressin was used as a control as the IV route provides 100% drug bioavailability and immediate administration into systemic circulation. Preliminary tests on a few piglets were conducted to determine the optimal ETT vasopressin doses. IN vasopressin doses were based on a study administering ~ 2.1–3.1IU/kg IN vasopressin in macaques^22^, but doses were increased following a lack of hemodynamic response to 4IU/kg IN vasopressin. All piglets, regardless of randomization, underwent insertion of an intraosseous needle during surgical instrumentation to ensure each piglet underwent similar surgical procedures. Arterial blood was collected before vasopressin administration (baseline), 1, 2, 3, 4, 5, and 10 min after vasopressin administration. Following final collection of blood, piglets were euthanized with an intravenous overdose of sodium pentobarbital (100 mg/kg).

## Data collection and analysis

Demographics of study piglets were recorded. Transonic flow probes, heart rate, and pressure transducer outputs, and Millar catheter were digitized and recorded with LabChart^®^ programming software (ADInstruments, Houston, TX). Blood samples were collected and centrifuged at 11,000 rpm for five minutes, plasma was then separated and stored at -80 °C. Concentrations of vasopressin were quantified using commercially available ELISA kits (K049-H1, Arbor Assays, Ann Arbor, Michigan, USA). Analysis of pharmacokinetic parameters was performed with the Non-Compartmental Analysis program of the SimBiology Model Analyzer app within MATLAB (MATLAB ver. R2023b, MathWorks, Natick, MA, USA).

The data was tested for normality (Shapiro-Wilk and Kolmogorov-Smirnov test) and compared using ANOVA for repeated measures using Tukey post-test for parametric and Dunn’s test for nonparametric comparisons of continuous variables, and Fisher’s exact test for categorical variables. The data are presented as mean (standard deviation - SD) for normally distributed continuous variables and median (interquartile range - IQR) when the distribution was skewed. *P*-values are 2-sided and *p* < 0.05 was considered statistically significant. Statistical analyses were performed with SigmaPlot (Systat Software Inc, San Jose, USA).

## Results

44 newborn mixed breed piglets (1–3 days of age, weighing 2.0 kg (± 0.23 kg)) were obtained on the day of experimentation. There were no differences in baseline parameters between IV and IO piglets (Table 1) or ETT and IN piglets within each route and vasopressin dose (Table 2). The sample size was reduced from five piglets per IN dose to three due the lack of hemodynamic response following drug administration.

**Table 1:** Baseline characteristics of intravenous and intraosseous doses.

	IV	IO			*p*-value
	Vaso 0.4 (*n* = 5)	Vaso 0.2 (*n* = 5)	Vaso 0.4 (*n* = 5)	Vaso 0.8 (*n* = 5)	
Age (days)	2 (2–3)	2 (2–3)	3 (3–3)	3 (3–3)	0.30
Weight (kg)	2.1 (1.9–2.2)	2.1 (1.7–2.1)	2.2 (2.2–2.4)	2.3 (2.2–2.3)	0.16
Sex (male/female)	3/2	1/4	2/3	3/2	0.58
pH	7.44 (7.44–7.44)	7.46 (7.45–7.50)	7.44 (7.42–7.47)	7.46 (7.41–7.47)	0.96
paCO_2_ (torr)	35.2 (34.3–39.7)	33.8 (32.4–36.3)	35.4 (32.6–35.5)	33.7 (33.3–33.9)	0.47
paO_2_ (torr)	82.7 (65.9–83.5)	90 (82.4–91.7)	87.8 (84.2–88.2)	82.2 (64.7–87.1)	0.20
Base excess (mmol/L)	0.7 (-0.2 ~ 2.9)	1.2 (-1.1 ~ 1.8)	-2.2 (-3 ~ 2.3)	0.6 (-3 ~ 2)	0.75
Lactate (mmol/L)	3.31 (3.14–3.33)	3.87 (3.77–4.32)	4.43 (4.3–5.21)	5.9 (4.77–7.13)	0.15
Hemoglobin (g/0.1 L)	7.4 (7.1–9)	6.7 (6.4–6.9)	7.3 (6.6–9.2)	7.3 (6.8–7.7)	0.23
Heart rate (bpm)	160 (152–166)	188 (182–188)	189 (159–196)	195 (175–200)	0.15
Mean arterial pressure (mmHg)	61 (59–65)	63 (61–73)	63 (61–65)	63 (57–75)	0.36
Carotid blood flow (mL/kg/min)	89 (52–96)	86 (77–100)	78 (73–88)	96 (92–103)	0.82
Cardiac output (mL/kg/min)	431 (407.36–495)	535 (530–575)	327 (287–523)	478.5 (333.5–530.5)	0.68
Ejection fraction (%)	45.61 (39.93–47.55)	34 (34–43)	28.7 (22–35.1)	37 (26.5–41)	0.36
Stroke volume (mL/kg/min)	2.69 (2.68–2.75)	3.03 (2.84–3.12)	2.13 (1.58–2.19)	2.21 (1.56–2.51)	0.18
dp/dt max (mmHg)	2690 (2157–2890)	2998 (2635–3777)	3323 (2623–3615)	3451 (2877.5–4227.5)	0.48
dp/dt min (mmHg)	-2776 (-3062~ -2022)	-4172 (-4792~ -3502)	-3608 (-4009~ -3171)	-3858.5 (-4616.5~ -3192)	0.09

Data are presented as median (IQR); IV, intravenous; IO, intraosseous; Vaso, vasopressin. P-values are comparing all IO vasopressin doses to IV vasopressin.

**Table 2:** Baseline characteristics of endotracheal and intranasal doses.

	ETT			*p*-value	IN			*p*-value
	Vaso 4 (*n* = 5)	Vaso 8 (*n* = 5)	Vaso 16 (*n* = 5)		Vaso 8 (*n* = 3)	Vaso 16 (*n* = 3)	Vaso 32 (*n* = 3)	
Age (days)	2 (1–2)	1 (1–2)	2 (1–2)	0.77	3 (2–3)	2 (1–2)	1 (1–3)	0.30
Weight (kg)	1.9 (1.8–1.9)	1.8 (1.8–2)	2 (1.8–2.1)	0.67	2.3 (2–2.3)	2.1 (1.8–2.2)	2 (1.9–2.1)	0.35
Sex (male/female)	2/2	4/1	4/1	0.59	2/1	3/0	2/1	0.63
pH	7.433 (7.427–7.448)	7.479 (7.449–7.492)	7.488 (7.429–7.5)	0.79	7.491 (7.411– 7.496)	7.531 (7.53–7.56)	7.484 (7.474–7.508)	0.06
paCO_2_ (torr)	34.5 (32.7–36.7)	33.6 (31.4–35.3)	32.7 (31.9–34.9)	0.80	30.8 (30.3–37.5)	29 (28.1–30.5)	31.3 (30.9–39)	0.33
paO_2_ (torr)	74.8 (69.2–79.1)	83.4 (77.6–86.1)	75.2 (71.1–79.9)	0.10	83.5 (70.1–92.9)	79.8 (73.3–90.4)	74.7 (61.4–83)	0.53
Base excess (mmol/L)	-1 (-1.2~ -0.1)	-0.1 (-0.6 ~ 2.6)	1.7 (-1.3 ~ 3.1)	0.80	0.2 (-0.8 ~ 0.3)	2.8 (0.8 ~ 3.8)	1.8 (-0.2 ~ 5)	0.23
Lactate (mmol/L)	4.25 (4.2–5.06)	3.81 (2.94–4.29)	4.57 (4.23–4.58)	0.78	4.25 (3.56–5.05)	4.26 (3.81–4.86)	4.31 (3.9–4.93)	0.98
Hemoglobin (g/0.1 L)	7.9 (7–8)	8.2 (8–9.6)	7.9 (7.8–9.7)	0.80	8.4 (6.8–8.6)	8.2 (8.2–9.8)	6.8 (6.5–8.1)	0.19
Heart rate (bpm)	163 (158–175)	137 (122–138)	167 (122–192)	0.43	166 (129–180)	156 (155–166)	143 (134–150)	0.42
Mean arterial pressure (mmHg)	67 (64–68)	53 (48–58)	53 (51–58)	**0.009**	64 (61–66)	61 (58–65)	60 (53–61)	0.22
Carotid blood flow (mL/kg/min)	83 (76–87)	56 (53–64)	63 (58–68)	0.30	62 (49–74)	70 (63–71)	60 (53–72)	0.66
Cardiac output (mL/kg/min)	384 (380–384)	290 (222–322)	320 (311–326)	0.32	259.05 (219.3–298.8)	514.7 (465–564.4)	375.2 (300.3–495)	0.09
Ejection fraction (%)	36 (27–37)	41 (35–45)	29 (28–33)	0.14	40 (28.7–51.3)	37.1 (32.6–41.6)	28.1 (24.5–59.7)	0.98
Stroke volume (mL/kg/min)	2.51 (1.72–2.9)	2.04 (1.92–2.1)	2.24 (1.81–2.43)	0.65	1.75 (1.7–1.8)	3.2 (3–3.4)	2.8 (2.1–3.3)	0.07
dp/dt max (mmHg)	2943 (2611–3742)	2278 (2212–2671)	2262 (2248–3224)	0.37	2727 (2239–3215)	2670.5 (2634–2707)	2530 (220.1–2584)	0.68
dp/dt min (mmHg)	-3526 (-4393~ -3380)	-3230 (-3339~ -3077)	-3275 (-3816~ -2587)	0.40	-3301 (-3684~ -2918)	-3832 (-4182~ -3482)	-3357 (-3540~ -3169)	0.40

Data are presented as median (IQR); ETT, endotracheal tube; IN, intranasal; Vaso, vasopressin.

### Intraosseous route

**Fig. 2:**
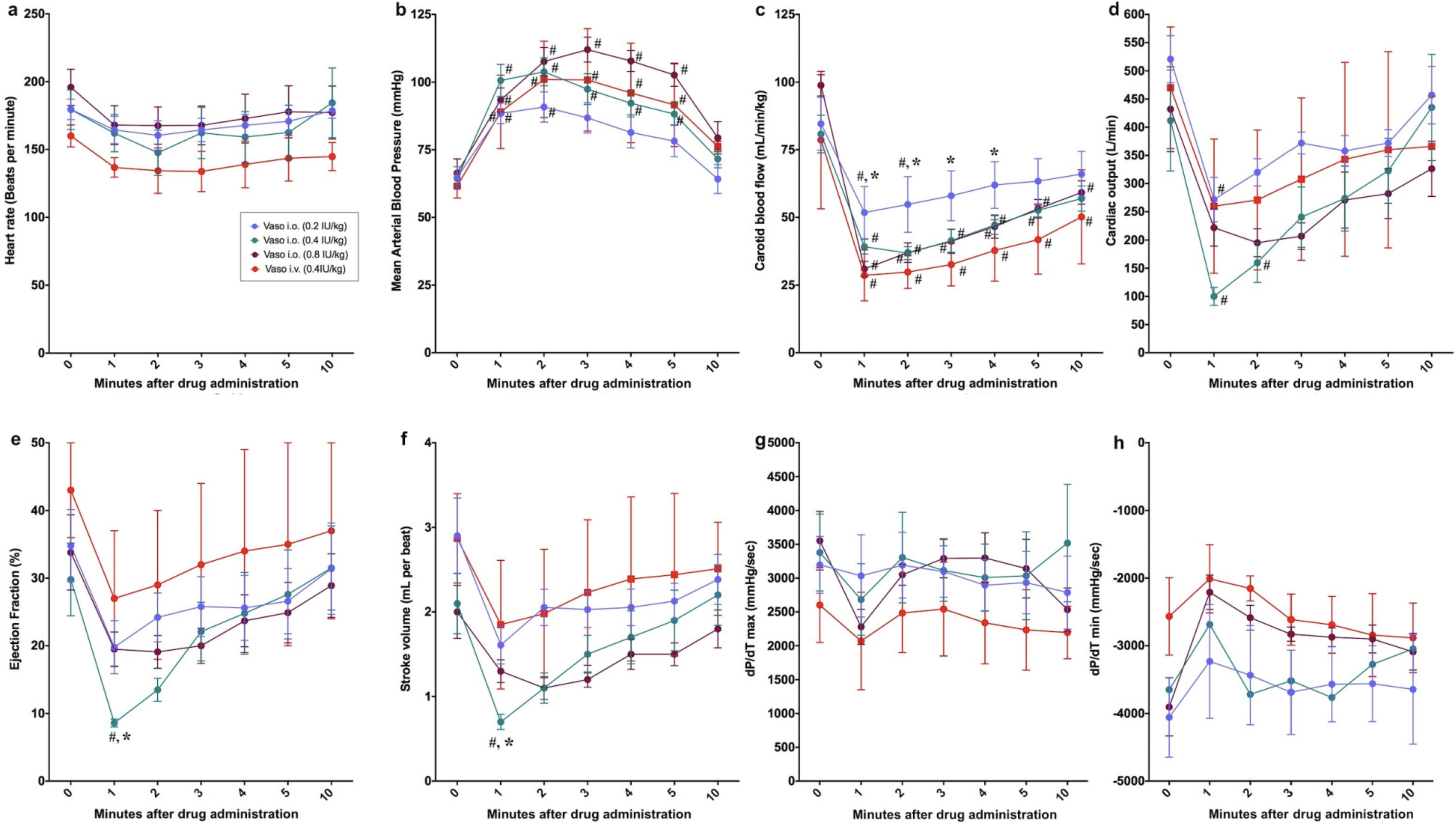
Changes in hemodynamic and cardiac function parameters following intraosseous vasopressin administration. Data are presented as mean (SD). Changes in (**a**) heart rate, (**b**) mean arterial blood pressure (**c**) carotid blood flow (**d**) cardiac output (**e**) ejection fraction (**f**) stroke volume, (**g**) dP/dT_max_, and (**h**) dP/dT_min_. # Significantly different from baseline; * Significantly different from 0.4IU/kg IV vasopressin (*p* < 0.05).

There were no significant changes in heart rate, dP/dT maximum or minimum in any IO-administered vasopressin dose compared to baseline values or IV vasopressin (Fig. 2a, g, h). MAP significantly increased for two minutes with 0.2IU/kg IO vasopressin, and five minutes with 0.4IU/kg IO vasopressin, 0.8IU/kg IO vasopressin, and IV vasopressin (Fig. 2b). Carotid blood flow significantly decreased five-, 10- and 10-minutes post-drug administration with 0.4IU/kg IO vasopressin, 0.8IU/kg IO vasopressin, and IV vasopressin, respectively, while carotid blood flow was decreased two-minutes after 0.2IU/kg vasopressin administration, but had comparable values to baseline from three to 10 min (Fig. 2c). Carotid blood flow was significantly higher after 0.2IU/kg IO vasopressin administration for four minutes compared to IV vasopressin (Fig. 2c). Cardiac output was significantly decreased with 0.2 and 0.4IU/kg IO vasopressin one- and two-minutes after drug administration, respectively (Fig. 2d). Compared to IV vasopressin, 0.4IU/kg IO vasopressin had significantly lower ejection fraction (8.4 (7.7–9.8)% vs. 23.3 (22.5–32.6)%, respectively, *p* = 0.042) and stroke volume (0.7 (0.63–0.91) mL/kg/min vs. 1.32 (1.31–2.63) mL/kg/min, respectively, *p* = 0.021) one minute after drug administration (Fig. 2e and f).

### Endotracheal route

**Fig. 3:**
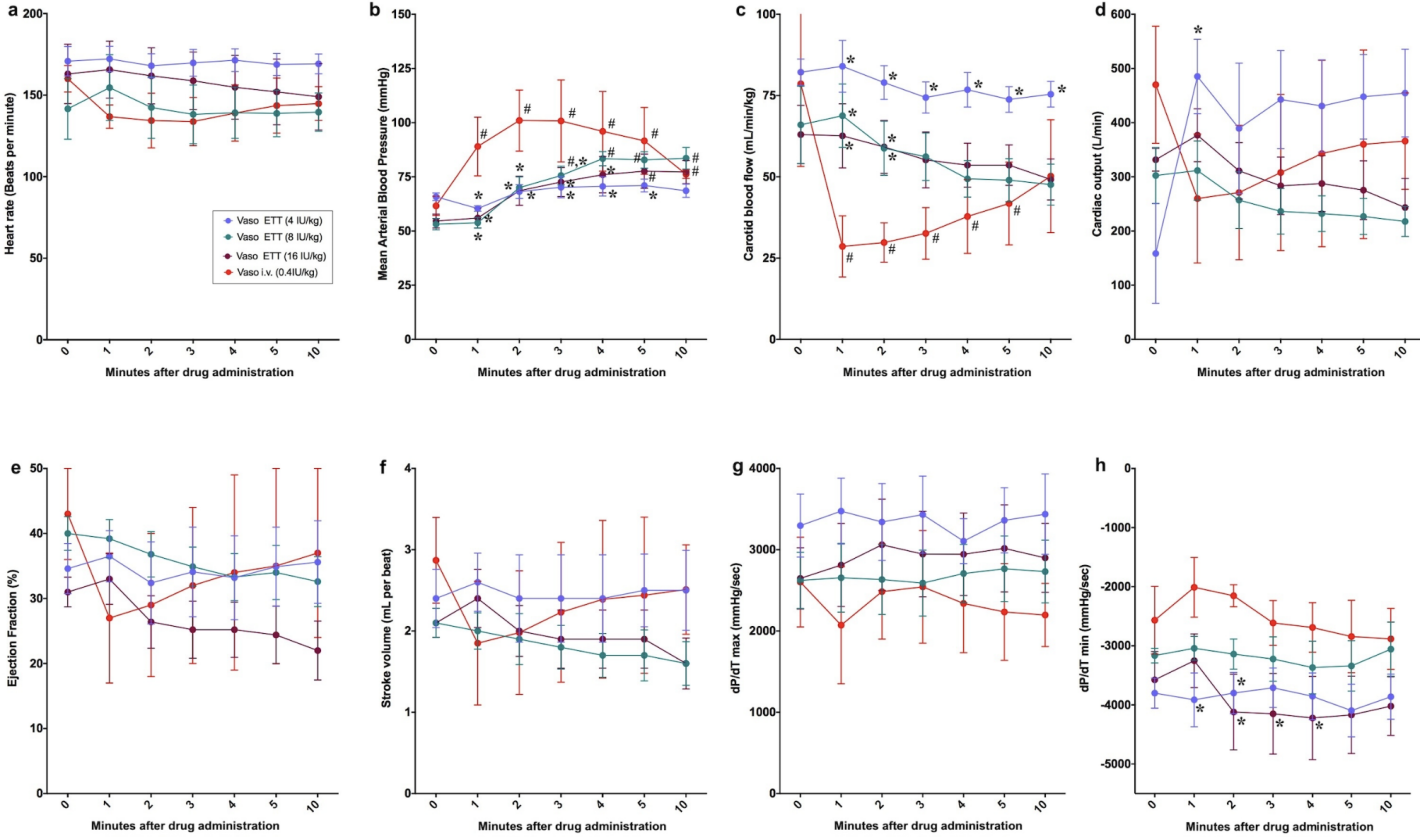
Changes in hemodynamic and cardiac function parameters following endotracheal vasopressin administration. Data are presented as mean (SD). Changes in (**a**) heart rate, (**b**) mean arterial blood pressure, (**c**) carotid blood flow, (**d**) cardiac output, (**e**) ejection fraction, (**f**) stroke volume, (**g**) dP/dT_max_, and (**h**) dP/dT_min_. # Significantly different from baseline; * Significantly different from 0.4IU/kg IV vasopressin at the concurrent time point (*p* < 0.05).

There were no changes from baseline in heart rate, carotid blood flow, cardiac output, ejection fraction, stroke volume, and dP/dT maximum or minimum with any vasopressin dose administered endotracheally (Fig. 3a, d–h). Significant differences in MAP and carotid blood flow were observed between IV vasopressin and all ETT vasopressin doses (Fig. 3b, c). Cardiac output was higher one minute after drug administration with 4IU/kg vasopressin compared to IV vasopressin (466 (394–558) mL/kg/min vs. 193.1 (170.3–365.8) mL/kg/min, respectively, *p* = 0.045) (Fig. 3d). dP/dT minimum was significantly lower with 4 and 16IU/kg vasopressin compared to IV vasopressin (Fig. 3h).

### Intranasal route

**Fig. 4:**
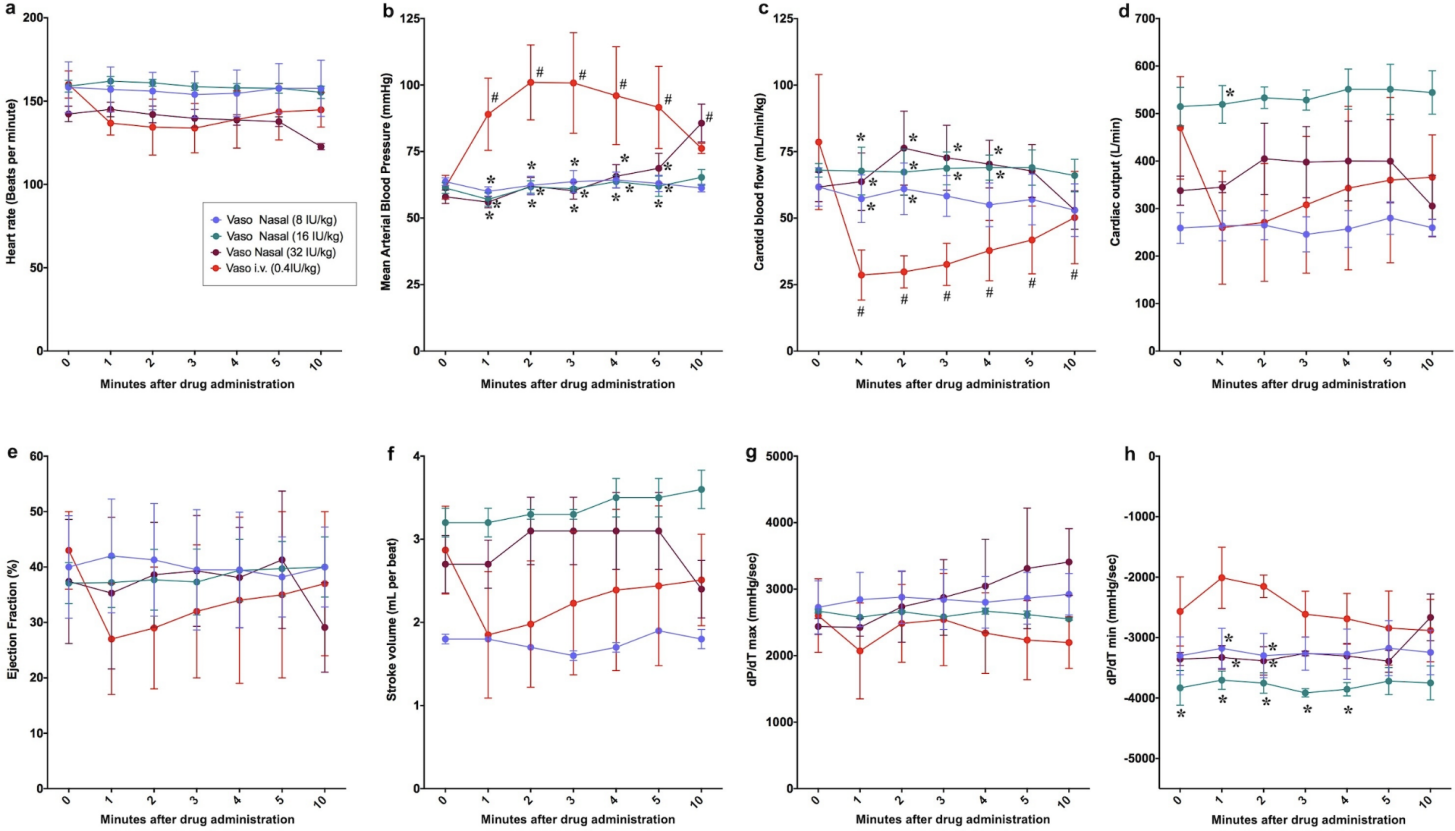
Changes in hemodynamic and cardiac function parameters following nasal vasopressin administration. Data are presented as mean (SD). Changes in (**a**) heart rate, (**b**) mean arterial blood pressure, (**c**) carotid blood flow, (**d**) cardiac output, (**e**) ejection fraction, (**f**) stroke volume, (**g**) dP/dT_max_, and (**h**) dP/dT_min_. # Significantly different from baseline; * Significantly different from 0.4IU/kg IV vasopressin at the concurrent time point (*p* < 0.05).

There were no changes in heart rate, carotid blood flow, cardiac output, ejection fraction, stroke volume, dP/dT maximum or minimum with any doses from baseline within the IN route (Fig. 4a, c–h). 32IU/kg vasopressin had significantly higher MAP 10 min after IN drug administration compared to baseline (82 (72–96) vs. 60 (53–61)), respectively, *p* = 0.016) (Fig. 4b). MAP and dP/dT minimum were significantly higher with IV vasopressin compared to all IN vasopressin doses, while carotid blood flow was higher with IN vasopressin doses compared to IV vasopressin (Fig. 4b, h, c).

### Plasma vasopressin concentrations

**Fig. 5:**
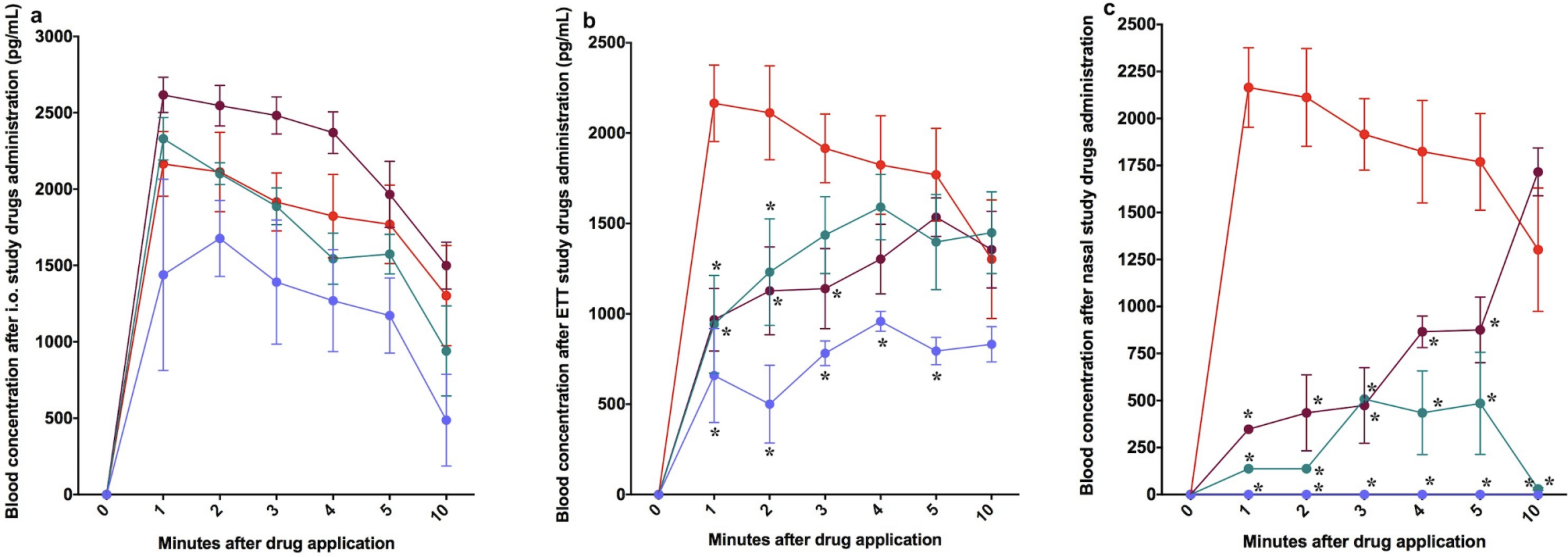
Plasma vasopressin concentrations following drug administration. Data are presented as mean (SD). Plasma vasopressin concentration after administration via (**a**) intraosseous (IO), (**b**) endotracheal (ETT), and (**c**) intranasal (IN) routes. In the IO group piglets were administered vasopressin 0.2IU/kg (blue circle), 0.4IU/kg (green circle), and 0.8IU/kg (purple circle. In the ETT group piglets were administered vasopressin 4IU/kg (blue circle), 8IU/kg (green circle), and 16IU/kg (purple circle). In the IN group, piglets were administered vasopressin 8IU/kg (blue circle), 16IU/kg (green circle), and 32IU/kg (purple circle). All doses were compared to 0.4IU/kg IV vasopressin (red circle). * Significantly different from 0.4IU/kg IV vasopressin at the concurrent time point (*p* < 0.05).

There were no differences in plasma vasopressin concentrations between any IO vasopressin dose (0.2, 0.4, or 0.8IU/kg) and IV vasopressin (Fig. 5a). Compared to IV vasopressin, 4, 8, and 16IU/kg ETT vasopressin had significantly lower plasma vasopressin concentrations two-, three-, and five-minutes after drug administration, respectively (Fig. 5b). 8 and 16IU/kg IN vasopressin had significantly lower plasma concentrations at all measured time points compared to IV vasopressin (Fig. 5c). 32IU/kg IN vasopressin achieved comparable plasma concentrations to IV vasopressin 10-minutes after drug administration (Fig. 5c).

### Pharmacokinetic parameters

**Fig. 6:**
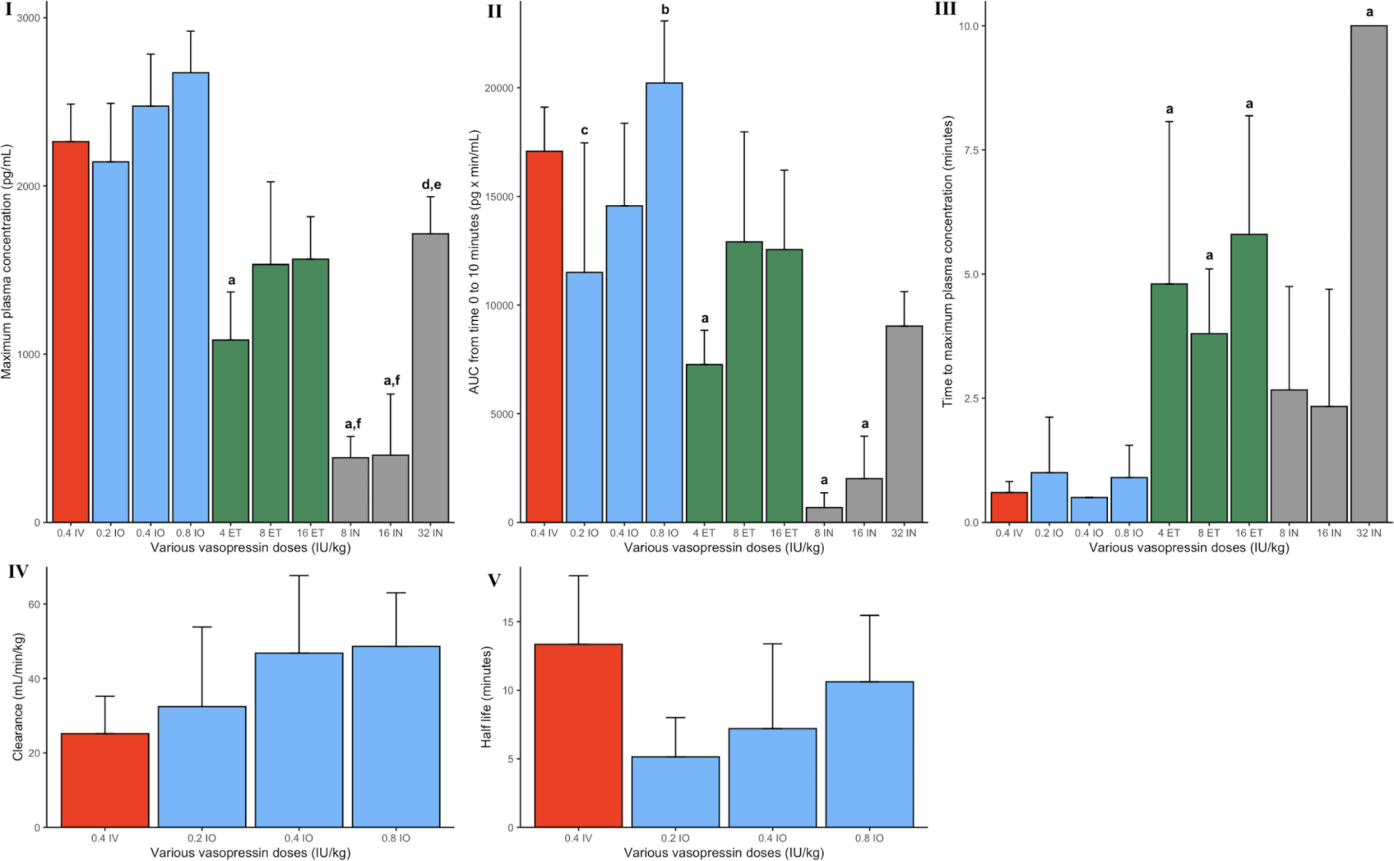
(**I**) Maximum vasopressin plasma concentration, (**II**) AUC (area under the curve) from baseline to 10 min, (**III**) time to maximum plasma concentration, (**IV**) clearance, and (**V**) half-life. Data are presented as mean (SD). IV, intravenous (red); IO, intraosseous (blue); ET, endotracheal (green); and IN, intranasal (grey). a, significantly different from the IV vasopressin group; b, significantly different from the 0.2IU/kg IO group; c, significantly different from the 0.8IU/kg IO group; d, significantly different from the 8IU/kg IN group; e, significantly different from the 16IU/kg IN group; f, significantly different from the 32IU/kg IN group (*p* < 0.05).

All parameters were comparable after IV or any IO vasopressin doses (Fig. 6I–V). *C*_*max*_ values were significantly lower with 4IU/kg ETT vasopressin (958 (44) pg/mL, *p* < 0.0001), 8IU/kg IN (384 (126) pg/mL, *p* < 0.0001), and 16IU/kg IN vasopressin (399 (363) pg/mL, *p* < 0.0001) compared to IV vasopressin (2,253 (63)). Significantly lower AUC_0–*t*_ compared to IV vasopressin (1,6213 (680) pg⋅min/mL) was observed with 4IU/kg ETT (7,264 (1,576) pg/min/mL, *p* = 0.02), 8IU/kg IN (675 (681) pg/min/mL, *p* < 0.0001) and 16IU/kg IN vasopressin (2,012 (1,952) pg/min/mL, *p* < 0.0001). *T*_*max*_ was significantly higher in all ETT vasopressin and 32IU/kg IN vasopressin piglets compared to IV vasopressin (Fig. 6III, *p* < 0.05). Elimination parameters of clearance and half-life could not be calculated for any ETT or IN doses as drug elimination had not yet begun.

## Discussion

There is a need to find the optimal vasoactive drugs during neonatal CPR^3^. While several studies have compared epinephrine and vasopressin during CPR in animal models, this paper is the first study to systematically assess the pharmacokinetic and pharmacodynamics of vasopressin administered via different routes. In our current study, we compared 0.4IU/kg IV vasopressin to vasopressin administered via IO, ETT or IN. The results of our study can be summarized as follows: (1) significant changes in hemodynamic parameters were most commonly observed with IO vasopressin (Fig. 2), (2) *C*_*max*_ and *T*_*max*_ were not different between IO or IV vasopressin doses (Fig. 6), and (3) ETT and IN routes resulted in poor drug absorption and hemodynamic response (Figs. 3, 4, 5, 6).

Administration of IO vasopressin resulted in significant changes in multiple hemodynamic parameters (Fig. 2). While there were no statistically significant changes in heart rate, dP/dT maximum or minimum in any IO vasopressin dose compared to baseline values or IV vasopressin, decreases in these parameters may have clinical relevance. The observed decrease in these parameters may be due to the administration of vasopressin during normoxic conditions compared to hypoxia. Boyle and Segel used an isolated working rat heart model to examine the effects of vasopressin during normoxic and hypoxic conditions and reported that vasopressin significantly decreases dP/dT maximum during normoxia but not hypoxia^23^.

There were no differences in pharmacokinetics between IO and IV vasopressin, which indicates that IO administration is as effective as IV administration for vasopressin delivery and absorption. In a pediatric swine model of ventricular fibrillation, Wenzel et al. compared 0.8IU/kg IV and IO vasopressin and reported similar plasma concentrations during CPR and the post–resuscitation period^24^. Similarly, Wimmer et al. compared humeral IO and IV administration of vasopressin in a hypovolemic swine model and reported no differences between mean *C*_*max*_, *T*_*max*_, serum concentrations during CPR, rates of ROSC, or survival^25^.

While there were no statistically significant differences in pharmacokinetic parameters between 0.4 or 0.8IU/kg IO vasopressin and IV vasopressin, 0.4IU/kg IO vasopressin had significantly lower ejection fraction one minute after drug administration while 0.8IU/kg IO vasopressin did not. This may result from the non-statistically significant lower baseline ejection fraction in 0.4IU/kg IO vasopressin piglets compared to 0.8IU/kg IO vasopressin piglets (Table 1). Nonetheless, we previously reported that 0.8IU/kg IV vasopressin resulted in significantly higher base excess and lactate concentrations four hours after resuscitation compared to 0.4IU/kg IV vasopressin^26^. Therefore, it appears that 0.4IU/kg vasopressin might be the preferable dose during neonatal resuscitation.

Significantly lower *C*_*max*_ with 4IU/kg ETT vasopressin and higher *T*_*max*_ in all ETT vasopressin doses compared to IV vasopressin indicates that the ETT route is an inefficient route of vasopressin administration. The significantly lower *C*_*max*_ and AUC with 8IU/kg and 16IU/kg IN compared to IV vasopressin indicates that IN administration of vasopressin is not an alternative for drug administration during neonatal CPR. ETT and IN administered vasopressors must be absorbed by epithelial cells and then transported into the systemic circulation, while IV and IO administered vasopressors are immediately deposited into circulation. This may account for the higher *T*_*max*_ values of ETT and IN compared to IV and IO routes. The lack of significant changes in hemodynamic parameters following IN and ETT administration of vasopressin may be due to its poor absorption into systemic circulation. Poor absorption may result from the rapid metabolism of vasopressin by peptidases located throughout the respiratory tract; however, without radioimmunoassay analyses we can only speculate if vasopressin was metabolized^27,28^. A study examining plasma concentrations of IN-administered 1-deamino-8-D-arginine vasopressin (DDAVP, a vasopressin analogue) in healthy adult males reported a bioavailability of approximately 10%, and peak plasma concentrations were observed 1–2 h after drug administration^29^. While elimination of vasopressin occurred within the IV and IO routes, there was no clearance after ETT or IN administration within 10 min, demonstrating delayed absorption. Therefore, elimination parameters of clearance and half-life could be determined for IV and IO vasopressin doses, but not ET or IN doses (Fig. 6 IV–V).

### Limitations

Based on the principle of reducing use in animal experiments, we discontinued the IN route after 12 piglets as we could not justify including further piglets without the prospect of achieving significant changes in hemodynamic parameters. Our neonatal model uses piglets that have already undergone the fetal to neonatal transition. Nevertheless, our findings are still clinically relevant as the distribution of cardiac output to vital organs (i.e. brain and heart) in the fetus and post-transitional neonate during asphyxia episodes are quantitatively similar^30,31^. Additionally, piglets were euthanized 10 min after drug administration; therefore, no comparisons could be made on potential long-term changes that may occur hours after drug administration. The only IN vasopressin dosage that produced any significant changes from baseline was 32IU/kg; however, tissue injury markers were not analyzed and requires further evaluation before clinical translation. Furthermore, our study examined various vasopressin doses in healthy post-transitional piglets, and will need to be replicated in a cardiac arrest model.

## Conclusion

The IO route provides rapid vasopressin delivery and absorbance, and analysis of pharmacokinetic parameters demonstrates comparable results as IV-treated piglets. In our study, IO vasopressin at 0.4IU/kg was most effective. Administration of vasopressin via ETT or IN resulted in unreliable absorption, no hemodynamics changes, and therefore might be unreliable during neonatal resuscitation.

## Electronic supplementary material

Below is the link to the electronic supplementary material.

Supplementary Material 1

## Data Availability

The datasets generated and analyzed for this study are available from the corresponding author (GMS), upon reasonable request.
